# Derivatization of Levoglucosan for Compound-Specific *δ*^13^C Analysis by Gas Chromatography/Combustion/Isotope Ratio Mass Spectrometry

**DOI:** 10.1155/2020/9571969

**Published:** 2020-07-26

**Authors:** Dongling Zhang, Swe Swe Aung, Yong Han, Yanlin Zhang, Yingjun Chen, Hongli Li, Feifei Yu, Yangjun Wang, Jialiang Feng, Wu Wang

**Affiliations:** ^1^Institute of Environmental Pollution and Health, School of Environmental and Chemical Engineering, Shanghai University, 200444 Shanghai, China; ^2^Yale-NUIST Center on Atmospheric Environment, Nanjing University of Information Science and Technology, 210044 Nanjing, China; ^3^Department of Environmental Science and Engineering, Fudan University, 200438 Shanghai, China

## Abstract

Levoglucosan is a thermal decomposition product of cellulose in particulate matter. *δ*^13^C value of levoglucosan could be used in studying the combustion mechanisms and chemical pathways. In order to introduce a minimum number of carbon atoms, based on the stereostructure of levoglucosan, a two-step derivatization method with methylboronic acid and MSTFA was developed and carefully optimized. The recommended reaction temperature is 70°C; the reaction time is 60 min for MBA and 120 min for MSTFA derivatization; and the molar ratio of levoglucosan : MBA : MSTFA is 1 : 1: 100 and 1 : 1: 120 and the reagent volume ratio of MSTFA : pyridine is between 1 : 3 and 1 : 4. The developed method achieved excellent reproducibility and high accuracy. The differences in the carbon isotopic compositions of the target boronate trimethysilylated derivative between the measured and calculated ranged from 0.09 to 0.36‰. The standard deviation of measured *δ*^13^C value of levoglucosan was between 0.22 and 0.48‰. The method was applied to particle samples collected from the combustion of cellulose at four different temperatures. *δ*^13^C values of levoglucosan in particle samples generated from a self-made combustion setup suggesting that combustion temperature play a little role on isotope fractionation of levoglucosan, although ^13^C enriched in levoglucosan during the combustion process.

## 1. Introduction

Levoglucosan (1.6-anhydro-*β*-D-glucopyranose) is a thermal decomposition product of cellulose and hemicellulose in biomass at temperatures above 300°C [1]. Pyrolysis of starch, sucrose, and neat glucose also produces levoglucosan [2–4]. Stable carbon isotope analysis is a valuable tool, because the stable isotope ratio of organic compounds depends on the isotope composition of the source, environmental conditions, and specific chemical pathways and mechanisms that lead to the formation of the compound. There have been reports measuring directly the *δ*^13^C values of levoglucosan as one of pyrolysis products of sucrose [2] and cellulose [5] using pyrolysis gas chromatography/isotope ratio mass spectrometry and levoglucosan in biomass burning aerosols by thermal desorption-2 dimensional gas chromatography/isotope ratio mass spectrometry [6].

**Figure 1:**
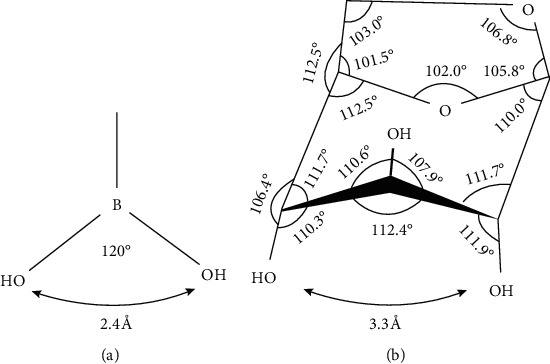
Stereostructures of methylboronic acid (a) and levoglucosan (b).

Theoretically, high-polarity levoglucosan has to be derivatized prior to gas chromatography/combustion/isotope ratio mass spectrometry (GC/C/IRMS) analysis. Methylboronic acid (MBA) has a planar, trigonal structure with the O-O distance 2.4 Å (Figure 1(a)). It contains only one carbon atom and readily condenses with two vicinal *cis*-diols and, therefore, has been used to determine natural ^13^C abundances of monosaccharides [7–10]. In our previous work, *δ*^13^C values of isoprene photooxidation products, 2-methyltetrols, in atmospheric aerosol were measured after MBA derivatization [11]. The advantage of MBA over other derivation reagents for the OH group, such as N,O-bis(trimethylsilyl)-trifluoroacetamide and acetic anhydride, is that it introduces a minimum number of carbon atoms.

Levoglucosan contains three hydroxyl groups in *trans*-ortho- and *cis*-meta-positions. The pyranose ring has the 1C chair conformation and is distorted by the formation of the anhydride (Figure 1(b)). C-1 and C-5 are compressed, and C-2 and C-4 are extended, resulting in the two *cis-*hydroxyls at C-2 and C-4 further apart and the angle at the pyranose ring oxygen smaller than in an unstrained pyranose ring system. The O-2–O-4 distance increases from 2.5 Å in the unstrained conformation to 3.3Å in levoglucosan and the angle at O-5 decreases from 112°to 102°, accordingly [12, 13]. Two OH groups of MBA might not react with levoglucosan. However, such distance could be adequate for the formation of a six-membered cyclic boronate ring, considering that the even longer O-O distance in*trans*-1, 2-dioIs (3.4 Å) can react with boronic acid [14]. Moreover, a six-membered ring (2,4-cyclic ester) formed from 2,4-diols with MBA in the chair conformation is accommodated in angle strain rather than a five-membered ring (2,3- or 3,4-boronate) in the boat conformation [13, 15]. In addition, Shafizadeh et al. reported that levoglucosan reacted quantitatively with benzeneboronic acid [16].

This study attempts to develop a MBA derivatization method for levoglucosan to analyze by the most commonly used GC/C/IRMS system. The remaining single OH was further derivatized by MSTFA (*N*-methyl-*N*-(trimethylsilyl)trifluoroacetamide). Multiresponse derivatization conditions were optimized, and *δ*^13^C fractionation in the derivatization reaction was assessed. Four standard levoglucosan with different *δ*^13^C values were used to determine the feasibility of the method. The isotopic fractionation effects were discussed based on the comparison of determined *δ*^13^C values and calculated values according to the stoichiometric mass balance. The method was applied to the particulate matter generated from combustion of microcrystalline cellulose at four temperatures.

## 2. Experimental Sections

### 2.1. Chemicals and Reagents

Levoglucosan was obtained from four suppliers: USP, USA (purity, 100%; **S1**); Sigma, USA (purity, 98%; **S2**); TRC, Canada (purity, 98%; **S3**); and Chiron, Norway (purity, 98%; **S4**). Methylboronic acid (purity, 97%) and methanol (GC grade) were purchased from CNW Technologies GmbH, Germany. Microcrystalline cellulose, *N*-methyl-*N*-(trimethylsilyl)trifluoroacetamide (MSTFA), and *myo*-inositol (purity, ≥99.5%) were from Sigma-Aldrich. Acetic anhydride was from Sinopharm, China, AR. Anhydrous pyridine (purity, 99%) was from Acros Organics, Geel, Belgium.

### 2.2. Instrumentation

GC/MS instrument consisted of a HP6890 GC (Agilent, USA) equipped with a DP-5MS (30 m × 0.25 mm i.d., 0.25 *μ*m film thickness), coupled to a HP5975MSD (Agilent, USA) quadrupole analyzer. Data were acquired and processed with ChemStation software. The oven temperature was initially 110°C, held for 2 min, 1°C min^−1^ up to 120°C, held for 5 min, then 5°C min^−1^ up to 180°C, held for 1 min, 30°C min^−1^ up to 300°C, and held for 1 min. The mass spectrometer was operated in EI at 70 eV and ion source temperature of 150°C. Full scan mode was used in the mass range *m/z* 40∼400.

GC/C/IRMS consisted of HP 6890 GC combined with an isotope ratio mass spectrometer (Isoprime, GV instrument), equipped with DP-5MS (30 m × 0.25 mm × 0.25 *μ*m). The temperature program started from 110°C, held for 2 min, 10°C min^−1^ up to 120°C, held for 5 min, then 5°C min^−1^ up to 300°C, and held for 5 min. The combustion furnace containing CuO catalyst and the reduction oven containing Cu catalyst were kept at 870°C and 580°C, respectively. The temperature of the interface between the GC and combustion furnace was set at 290°C. An external CO_2_ reference gas (*δ*^13^C = −29.10 ‰) was used to obtain highly accurate isotopic compositions. The reproducibility and accuracy of carbon isotopic analyses were evaluated routinely every day using 10 laboratory isotopic standards (C_12_, C_14_, C_16_, C_18_, C_20_, C_22_, C_25_, C_28_, C_30_, and C_32_*n*-alkanes supplied by Indiana University) with predetermined isotopic values (−31.89, −30.67, −30.53, −31.02, −32.24, −32.77, −28.49, −32.11, −33.05, and −29.41‰, respectively). Standard protein casein (*δ*^13^C = −26.98‰) was analyzed 10 times and used as laboratory standards.

EA/IRMS was performed as follows: capsules containing weighed samples were placed in the CE EA1112 C/N/S analyzer and burned at 960°C in an O_2_ atmosphere in a combustion tube. Combustion gases were swept through a reduction oven and entered a GC column where CO_2_ was separated from other gases. Then, the CO_2_ passed through a Conflo III interface (Finnigan) and entered a DELTA^plus^ XL mass spectrometer (Thermo Finnigan MAT, Bremen, Germany) where it was compared with the reference CO_2_of known *δ*^13^C value (−29.10‰, calibrated against the NBS-22 reference material with *δ*^13^C value −29.7‰). During every batch of analyses, an empty tin capsule was analyzed as the blank to check the background, and the protein casein (*δ*^13^C = −26.98‰) was used to evaluate the reproducibility and accuracy. The standard deviation of analysis and the deviation between the measured data and the predetermined data were both 0.13‰.

Combustion experiments were carried out in air flow with a homemade equipment. For the details, see [17]. Briefly, the whole set-up consists of a quartz tube (*φ* = 60 mm; height = 1200 mm) furnace, a dilution sampling system ((FPS-4000, Dekati Inc, Finland), two PM samplers using quartz fiber filters (90 mm, Whatman).One of the advantages of this equipment is that only a few grams of material is need for one combustion. In this case, 2 g of microcrystalline cellulose was sufficient to produce necessary amount of levoglucosan for GC/C/IRMS measurements for each combustion. No mass loss was observed for microcrystalline cellulose before and after burning when the temperature was 200°C. Therefore, the temperature was set at 300, 400, 500, and 600°C, respectively. For each temperature, combustion took place twice, and four filter samples were collected. Quartz fiber filters were organic free and stored at −20°C after sample collection.

### 2.3. Optimal Derivatization Conditions

**Figure 2:**
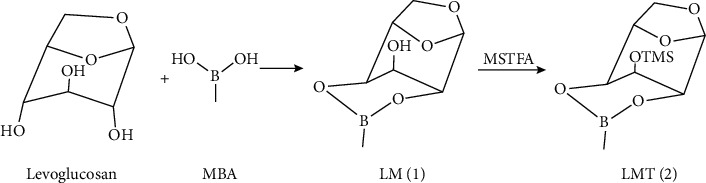
Derivatization reaction of levoglucosan with MBA and MSTFA.

A two-step MBA + MSTFA derivatization reaction of levoglucosan is shown in Figure 2. The first derivatization product levoglucosan methylboronate is abbreviated as LM or compound **1**, while the second one levoglucosan methylboronate trimethylsilyl ether as LMT or compound **2**. The optimal derivatization conditions were applied for the whole study. Initially, 240 *μ*L of a solution of levoglucosan (approximately 1 mg levoglucosan in 10 mL methanol) was dried under nitrogen in a 2 mL amber reaction vial, and then 90 *μ*L of a solution of 1 mg MBA in 10 mL anhydrous pyridine was added. The mixture was allowed to react at 70°C for 60 min. After cooling down to the room temperature, 30 *μ*L MSTFA in 100 *μ*L anhydrous pyridine was added. The vial was then capped, vortexed, and heated in a dry block heater at 70°C for120 min. Yields of LMT (**2**) were monitored by GC/MS.

For combustion filter samplers, the amount of levoglucosan was determined by GC/MS following our previous work usingmethyl-D-xylanopyranoside (MXP, Sigma) as the internal standard [18]. Then, the optimal derivatization conditions were applied for GC/C/IRMS analyses.

## 3. Results and Discussion

The signal to noise (S/N) was obtained from six replicate measurements of low concentration (1 *μ*g/mL) of levoglucosan. Limit of detection (LOD) was 0.32 mg/ml (S/N = 3), and limit of quantification was 2.5 mg/mL (S/N = 10) for the GC/MS analysis. Approximately 24 *μ*g of levoglucosan was needed for a single GC/C/IRMS measurement. Each sample was measured at least three times.

### 3.1. GC/MS Data

**Figure 3:**
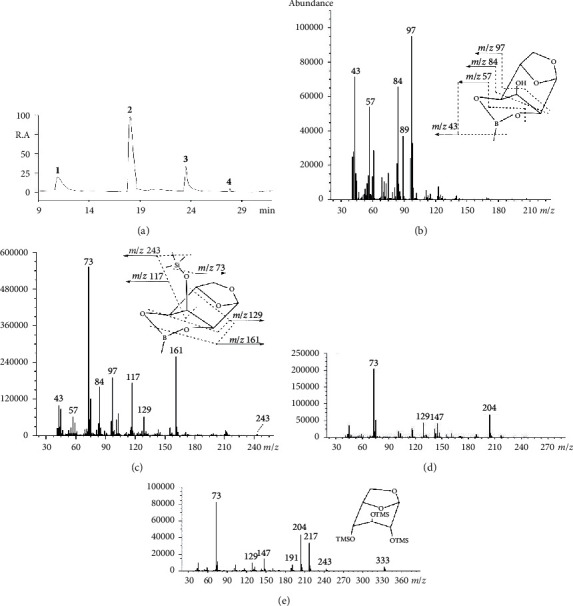
GC/MS chromatogram of levoglucosan derivatized by MBA + MSTFA (a) and the corresponding mass spectra of (b) compound **1** (LM), (c)compound **2** (LMT), (d) unknown by-product compound **3**, and (e) compound **4** (LT), together with the proposed fragmentation patterns for **1** (inset in b) and **2** (inset in c), under optimal derivatization conditions: 70°C; 60 min for MBA; and 120 min for MSTFA derivatization, respectively; levoglucosan : MBA : MSTFA = 1 : 1 : 120; MSTFA : pyridine (v : v) = 1 : 3.

Taking the abovementioned stereostructural information into account, indeed, product LM (compound **1**; Figure 2) formed after the reaction mixture is being heated. According to the reaction equation, levoglucosan reacts with equal mole of MBA. A molar ratio 1 : 1 of levoglucosan : MBA was applied and boronate derivative LM was obtained as the single product. This may be attributed to the formation of a more stable 2,4-cyclic ester six-membered ring than 2,3- or 3,4-boronate five-membered ring in monosaccharides or polyolsin which cases MBA to be in excess [7–11]. Unfortunately, this peak was broad (Figure 3(a)), due to the remaining free OH interaction with the solid phase of DB-5MS column. Medium polar DB-17 and DB-1701 columns were used to test the LM behavior; the peak shape from both columns was tailing with limited improvement. And, it did not get any better with polar DB-225 column either; even worse, the baseline elevated. Therefore, the remaining OH group of LM (**1**) has to be further derivatized.

In the second step, when using MSTFA + 1%TMCS to derivatize the remaining free OH, 2,4-cyclic boronic ester ring (LM, compound **1**; Figures 2, 3(a), and 3(b)) opened and was substituted by trimethylsilyl (TMS) groups leading to the formation of the fully TMS-derivatized product LT (compound **4**; Figures 3(a) and 3(e)). To reduce the derivatization efficiency, MSTFA without TMCS catalyst was applied. Multiple peaks were observed (Figure 3(a)), including the target product LMT (compound **2**; Figures 2, 3(a), and 3(c)) and by-product compounds **3** and **4**. More by-products could be formed and eluted out at the retention time around **3** (polarity between **2** and **4**) depending on reaction conditions, as seen by other studies [8, 9]. Therefore, a multiresponse optimization for the second derivation was carried out in order to obtain both maximum yield of LMT (**2**) and the minimum number of by-products. The parameters, namely, reaction temperature, duration, molar ratio of levoglucosan : MSTFA, and solvent composition were optimized. Each independent variable was investigated at five or six levels. All measurements were done in triplicate. The responses were peak areas.

Figure 3 displays the GC chromatogram of the derivatization reaction under optimal condition (a), the mass spectra of products (b–e), and the possible fragmentation patterns for LM (**1**) and LMT (**2**) (insets). This is the first report of GC/MS data for boronate LM and trimethylsilylate boronate levoglucosan LMT, and no authentic surrogates are available for structural conformation. Short discussion of mass spectrometric identification of LM and LMT are given below.

**Scheme 1:**
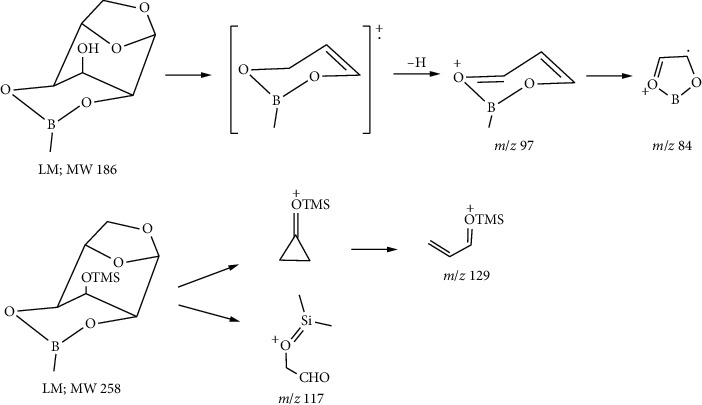
Plausible fragmentation pathways for characteristic ions of LM and LMT in EI.

The cyclic boronate group is usually stable and yields ions of major intensity, for example, the base peak *m*/*z* 97 in levoglucosan MBA derivative LM (**1**) which was formed by the loss of a hydrogen atom from *m*/*z* 98 (Figure 3(b) and the inset), demonstrating a six-membered boronate ring [13, 19]. It further rearranged to *m*/*z* 84 by eliminating the CH group Scheme (1). The boron-containing fragmentation ion at *m*/*z* 57 was formed by the cleavage of the boronate ring, combining the loss of CH_2_ resulting in *m*/*z* 43 (Figure 3(b) and the inset).

The base peak *m*/*z* 73 corresponding to the ion of Me_3_Si^+^ in target product LMT (**2**) supported a monoboronate cyclic ester with one trimethylsilyl substituent (Figure 3(c) and the inset). The peak pattern and ion composition of *m*/*z* 43, 57, 84, and 97 were the same as those of LM (**1**), suggesting a strong structural connection of these two compounds. In high mass range, a tiny *m*/*z* 243 corresponded to the loss of a methyl group, as often seen in TMS ethers (Figure 3(c) and the inset). Another high mass range ion *m*/*z* 161 was tentatively attributed to the cleavage of pyranose ring at C-2 and C-4 and elimination of methyl boryl fragment (Figure 3(c) and the inset), in contrast to the greater stability of boron-containing ions than the corresponding trimethylsilylated compounds in boronate trimethylsilyl ether derivatives of carbohydrates [19]. This could be explained by the angle distorted at boron, presumably as much as 10°, caused by anhydro ring in the present conformation (Figures 1 and 2). The ion at *m*/*z* 129 may involve a cyclization of C-2 and C-4 and subsequent rearrangement [20]. The ion at *m*/*z* 117 formally corresponds to CH_3_^+^CHOTMS. However, there is no terminal methyl group in LMT. It was suggested that *m*/*z* 117 resulted from rearrangement of the TMS group. Possible fragmentation pathways for ions at *m*/*z*117 and 129 are presented in Scheme 1.

Both ions at *m*/*z* 147 and 204 in compound **3** indicated more than one TMS group occurred in the molecule (Figure 3(d)). Their structures have been well documented as TMSO^+^ = Si(CH_3_)_2_ and TMSOCH = CHOTMS, respectively [16, 19]. Other major ions, such as *m*/*z* 73 and 129, were the same as those in LMT (**2**). It is worth noting that the absence of characteristic ions for boronate derivative at *m*/*z* 43, 57, 84, and 97 was the further evidence for di-TMS structure. Besides, the by-products in this study including the derivative (**3**) had retention time between LMT (**2**) and fully trimethylsilylated derivative LT (**4**), which was understandable because TMS was generally less polar than boronate. Structure of LT (**4**) was confirmed by comparing the spectrum with literature data (Figure 3(e)) [21].

### 3.2. Optimization of Derivatization Conditions

#### 3.2.1. Reaction Temperature

Derivatization reaction was performed under six temperatures, i.e., room temperature (25°C), 60, 70, 80, 90, and 100°C, (Table 1). No LMT (**2**) was formed at room temperature after reacting for 3 hours. The production of LMT (**2**) increased from 60°C to 70°C and decreased at 80°C. The reaction mixture was totally vaporized after 20 min at 90°C, and the production of LMT (**2**) was precarious and became undetectable at 100°C. Gross and Glaser [8] also observed 100°C was too hot in monosaccharide MBA derivatization and slightly larger product peaks at 80°C than 60°C but eventually used 60°C for both MBA and BSTFA derivatizations, so did van Dongen et al. [7] and our previous work with tetrols [22, 23]. Jackson et al. simply derivatized glucose with alkylboronic acid at 80°C and successive BSTFA at 60°C [9]. No data are available for 70°C. Based on the result shown in Table 1, the optimal reaction temperature for the MSTFA derivatization was 70°C. For practical reason, the temperature for the first step was adjusted from 60°C to 70°C, as the difference of LM peak areas at these two temperatures was not significant.

#### 3.2.2. Reaction Time

**Figure 4:**
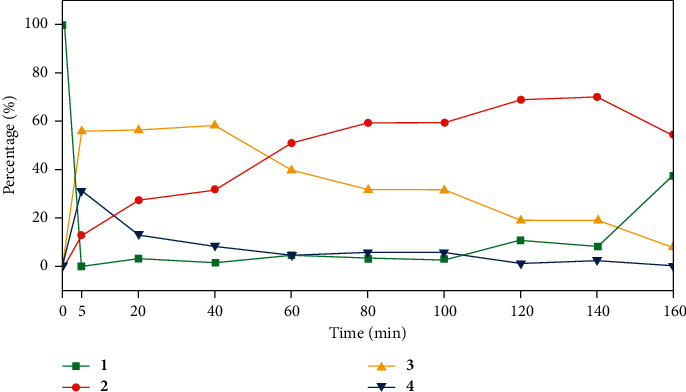
Percentage of peak areas of starting compound LM (**1**) and product compounds **2**–**4** over the total peak area evolving with reaction time. Derivatization conditions: levoglucosan : MBA : MSTFA = 1 : 1 : 120, MSTFA : pyridine (v : v) = 1 : 3.

The effect of reaction time was evaluated by taking 120 molar equivalents of MSTFA to levoglucosan and reacting at 70°C for 5, 20, 40, 60, 80, 100, 120, 140, and 160 min, respectively.  Figure 4 shows that compound **1** (LM) dropped dramatically in 5 min after the addition of MSTFA to the first step reaction mixture. The target compound LMT (**2**) increased steadily over time, while by-products **3** and **4** declined gradually after 40 min and 20 min, respectively. There was no significant improvement in the yield of LMT for the reaction time longer than 120 min (Figure 4). As discussed above, although levoglucosan has no vicinal *cis*-diols, the O-2-O-4 distance (3.3 Å) is in the range of the O-O distance of MBA (2.4 Å). LM (**1**) does not seem more difficult to form than monosaccharide or polyol MBA derivatives in terms of reaction temperature and duration [7–11].

However, significant difference in the behavior of levoglucosan and monosaccharides was revealed in the second step derivatization. Firstly, it took 120 min for LM (**1**) to get converted into LMT (**2**), compared with only 5 min needed for monosaccharide boronates to get converted to TMS derivatives [7, 8]. This can be explained by the steric hindrance in which the hindered nature of HO-3 is a result of the proximity of the semirigid 1,6-anhydro ring in levoglucosan. Secondly, as seen from Figure 4, within the first 5 min after the addition of MSTFA, the first step derivative product LM transformed almost completely, indicating a tendency for the boronate ring to break down to form TMS derivatives (compounds **3** and **4**). The instability of the boronate may be attributed to the formation of the six-membered boronate ring which generated steric hindrance [19]. Thirdly, it implied that the target product LMT (**2**) could partially be formed from restructuring of compounds **3** and **4** under the reaction condition.

#### 3.2.3. Effect of Reagent: Molar Ratio of Levoglucosan : MSTFA

It has been documented that MSTFA has advantage over BSTFA as TMS donor owing to the higher volatility of MSTFA itself and its by-product *N*-methyltrifluoroacetamide [24]. BSTFA and its by-products including mono-(trimethylsilyl)trifluoroacetamide and trifluoroacetamide may result in interference in chromatograms. Here, MSTFA was chosen for TMS derivatization. However, the amount of MSTFA was found to have effect on the formation of LMT in the reaction. Five molar ratios of levoglucosan to MSTFA were evaluated.

**Figure 5:**
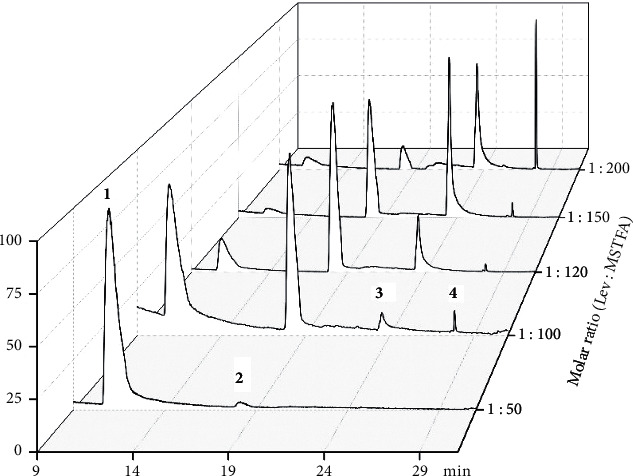
TICs of levoglucosan derivatized by MBA and MSTFA at five molar ratios of levoglucosan : MSTFA (levoglucosan : MBA = 1 : 1).

Figure 5 demonstrates that levoglucosan : MSTFA (molar ratio) at 1 : 50 did not work: LM (**1**) barely reacted and little target product LMT (**2**) formed. With the increase in molar ratio to 1 : 100, **1** was converted partially, **2** was formed significantly, and by-products **3** and **4** started to appear. The TIC at molar ratio 1 : 120 gave the best distribution of starting compound LM (**1**), target product LMT (**2**), and by-products **3** and **4**. Adding more MSTFA resulted in the decrease of not only LM but also LMT; in the meantime, significant increase of by-products **3** (1 : 150) or **4** (1 : 200) was observed, which was undoubtedly due to too high amount of MSTFA.

In general, with the increase of MSTFA (molar ratio from 1 : 50 to 1 : 120), LM (**1**) got converted to the target compound LMT (**2**) gradually with no (1 : 50) or ignorable (1 : 100) amount of by-products **3** and **4**. The peak area of LMT was the largest when levoglucosan : MSTFA was 1 : 120.Further increase of MSTFA to 200 molar equivalents did not favor the formation of **2** any longer. The optimal molar ratio of levoglucosan : MSTFA ranged between 1 : 100 and 1 : 200.

#### 3.2.4. Effect of Solvent : Volume Ratio of MSTFA : Pyridine

Pyridine has the advantage that it acts as an acid scavenger removing H^+^ produced during TMS derivatization which would otherwise contribute to GC column stationary phase deterioration over time. In this experiment, dry pyridine dissolved the sample and derivatization products as solvent, facilitated effective silylation, and prevented the hydrolysis of the products once exposed to moisture. However, the extent of conversion and the effectiveness of derivatization were found to be dependent on the amount of pyridine added during the experiments.

**Figure 6:**
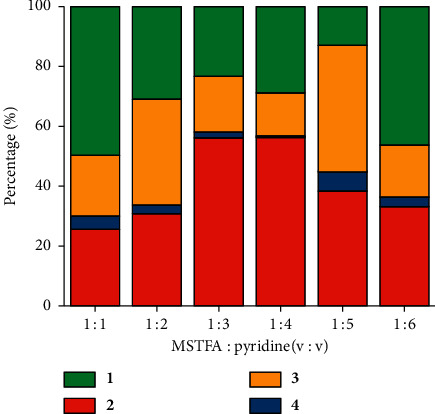
Percentage of individual peak area over the total peak area under six levels of MSTFA : pyridine (v : v) (levoglucosan : MBA : MSTFA = 1 : 1 : 120).

Figure 6 demonstrates that the amount of solvent played a role in the derivatization procedure. The yield of target product LMT (**2**) was highest when MSTFA : pyridine (v : v) ratio was between 1 : 3 and 1 : 4 (Figure 3(a)). Under other ratios, for example, MSTFA : pyridine = 2 : 1 and 1 : 6, a few more by-products eluted out around the retention time of compound **3**, sometimes with noticeable elevation of the base line (not shown). The mass spectra of those compounds were similar to those of compound **3** characterized by major fragmentation ions at *m*/*z* 73, 129, and 204, none of them corresponding to boronate (*m*/*z* 43, 57, 84, 97; Section 3.1). These clearly indicated that (1) by-products were TMS-related bearing no boronate ring; (2) by-products were less polar and therefore eluted after LMT (**2**) which contained a boronate ring from the nonpolar column; (3) the amount of pyridine affected the derivatization effectiveness of MSTFA. The optimal MSTFA : pyridine (v : v) ratio could be from 1 : 3 to 1 : 4.

### 3.3. Isotopic Fractionation Effects

**Figure 7:**
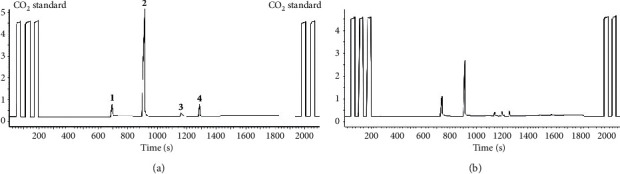
GC/C/IRMS chromatograms of MBA + MSTFA derivatization of standard levoglucosan (a) and the extracted particle sample collected from combustion of microcrystalline cellulose at 400°C (b), respectively.

Figure 7(a)shows the GC/C/IRMS chromatogram of levoglucosan derivatized by MBA and MSTFA using the optimized method. There were four peaks, same as the chromatogram obtained from GC/MS (Figure 3(a)), as expected, because the same GC column was used. The highest peak corresponding to LMT (**2**) was base line separated and narrow, qualified for isotope measurements.

The theoretical *δ*^13^C values of LMT can be calculated according to stoichiometric mass balance (equation (1) and reflect the relative contributions of carbon from levoglucosan, MBA, MSTFA, and their respective *δ*^13^C values (*δ*^13^C of the TMS group for MSTFA). To determine the isotopic composition of the TMS group, a *myo*-inositol standard with known *δ*^13^C value was trimethylsilylated with the same batch of MSTFA. Then, the *δ*^13^C value of TMS group was calculated by determining the carbon isotopic composition of the derivatized standard:

(1) $$\begin{array}{c} \delta^{13}{\text{C}}_{\text{LMT}}=f_{\text{levoglucosan}}\delta^{13}{\text{C}}_{\text{levoglucosan}}+f_{\text{MBA}}\delta^{13}{\text{C}}_{\text{MBA}}+f_{\text{TMS}}\delta^{13}{\text{C}}_{\text{TMS}}, \end{array}$$

where *f*_levoglucosan_, *f*_MBA_, and *f*_TMS_ are the molar fractions of carbon in LMT arising from underivatized levoglucosan, MBA, and TMS, respectively. Here, *f*_levoglucosan_ = 3/5, *f*_MBA_ = 1/10, and *f*_TMS_ = 3/10.

The stable carbon isotopic compositions obtained for levoglucosan and LMT are displayed in Table 2. The measured *δ*^13^C value for LMT was compared with the predicted one by equation (1). The predicted and measured *δ*^13^C values of LMT agreed well with each other and were within the precision limits of 0.5‰ for the GC/C/IRMS system.

According to Rieley's discussion on kinetic isotope effect [25], when a bond containing the carbon atom under consideration is changed in the rate-determining step, the primary isotopic effect is the most significant. If no carbon bond changed in the rate-determining reaction or if no carbon-containing bond is involved in this step, there should not be primary isotope effect on the *δ*^13^C value. In this work, two hydroxyl groups of levoglucosan reacted with one molecule of MBA and the remaining free hydroxyl group was trimethylsilylated. No carbon bond changed in the reaction. The difference between measured and calculated *δ*^13^C values of LMT ranged from 0.09 to 0.36‰ (Table 2). Accuracy was well within the isotope technical specification (±0.5‰). It should be pointed out that the analytical error of the calculated data for underivatized levoglucosan (usually expressed as the standard deviation, S) could be calculated by using the following equation:

(2) $$\begin{array}{c} S_{\text{levoglucosan}}^{2}={\left(\frac{1}{f_{\text{levoglucosan}}}\right)}^{2}{\text{S}}_{\text{LMT}}^{2}+{\left(\frac{f_{\text{MBA}}}{f_{\text{levoglucosan}}}\right)}^{2}{\text{S}}_{\text{MBA}}^{2}+\;{\left(\frac{f_{\text{TMS}}}{f_{\text{levoglucosan}}}\right)}^{2}{\text{S}}_{\text{TMS}}^{2}, \end{array}$$

where *f*_levoglucosan_, *f*_MBA_, and *f*_TMS_ are the same as those in equation (1), and S_levoglucosan_, S_MBA_, and S_TMS_ are the analytical standard deviations of levoglucosan, MBA, and TMS groups, respectively. In this work, S_LMT_, S_MBA_, and S_TMS_ were 0.13–0.29‰, 0.11‰, and 0.03‰, respectively. The standard deviation of the measured *δ*^13^C value of levoglucosan ranged from 0.22 to 0.48‰. The results imply that the method is promising and introduces no isotopic fractionation in the derivatization process, as confirmed in Table 2. The stable carbon isotopic determination acquired high precision and good reproducibility.

### 3.4. Application to Combustion Particle Samples

Figure 7(b) displays a typical GC/C/IRMS chromatogram of particle sample collected from the combustion of microcrystalline cellulose. The optimal derivatization conditions were applied after the quantification of levoglucosan in filter samples. Target compound LMT (**2**) was the most intensive peak and in good separation, similar to the chromatogram obtained from standard levoglucosan (top). This was also consistent with the GC/MS data which showed that levoglucosan was the only product for all four temperatures, as expected, since it is certain that levoglucosan is the major particle product of cellulose heated either under oxygen (combustion) or no oxygen (pyrolysis).  Table 3 presents *δ*^13^C values of microcrystalline cellulose and its combustion particle product levoglucosan under four temperatures. ^13^C was enriched in levoglucosan during the combustion process for all four temperatures, presumably resulting from the ^13^C depletion in gas phase products, as other studies observed [2, 5, 6]. On the other hand, the variations in temperature did not seem to have a significant effect on the level of enrichment of ^13^C. The standard deviation of enrichment for four temperatures was 0.15‰, suggesting combustion temperature (300–600°C) plays a little role on isotope fractionation of levoglucosan.

## 4. Conclusions

This is the first attempt to develop a derivatization method for levoglucosan to be amenable for widely used GC/C/IRMS analysis. The structures of two derivatization products LM and LMT were characterized based on the mass spectrometric data. Plausible fragmentation pathways were provided for the major ions. The results highlight the importance of optimizing derivatization conditions. Accurate *δ*^13^C value of the TMS group other than whole MSTFA molecule was also crucial. The carbon isotope data were reproducible, and no isotopic fractionation occurred during MBA + MSTFA derivatization. The method was successfully applied to the particle samples collected from combustion of microcrystalline at 300, 400, 500, and 600°C. ^13^C was enriched in levoglucosan by an average of 1.61‰ compared with the microcrystalline cellulose during combustion for four temperatures. And, the level of enrichment in levoglucosan was found to be irrelevant to the combustion temperature.

The method may be applicable for other mechanism researches, for example, to investigate *δ*^13^C values of levoglucosan in different particle sizes formed from the combustion of cellulose. Nevertheless, it may not be suitable for samples with insufficient levoglucosan concentrations (in this study, approximately 24 *μ*g of levoglucosan was needed for a single GC/C/IRMS measurement) and those with other compounds that react with MBA, such as mannosan and galactosan in atmospheric aerosols. Further work needs to be done to meet the requirements for more complex samples. Recently, Blees et al. reported methylation of levoglucosan catalyzed by dimethyl sulfide for oxygen isotope measurement by gas chromatography/pyrolysis isotope ratio mass spectrometry [26]. Since methylation of levoglucosan introduces only three carbon atoms, it could be an alternative. In addition, high-performance liquid chromatography could be used to detect levoglucosan directly [27, 28], and it might be worthwhile to couple HPLC to IRMS to avoid derivatization.

## Figures and Tables

**Table 1:** Percentage of peak area of target product LMT (**2**) over the total peak area at six temperatures.

Temperature (°C)	LMT (%)
Room temperature	—
60	36
70	70
80	15
90	—
100	—

**Table 2:** Measured and predicted stable carbon isotopic compositions of levoglucosan and LMT.

Suppliers	*δ* ^13^C^a^			
	Measured levoglucosan^b,c^	Measured LMT^b,d,e^	Calculated LMT ^f^	Δ^g^
S1	−15.65 ± 0.08 (*n* = 3)	−24.53 ± 0.13 (*n* = 3)	−24.32	0.21
S2	−14.45 ± 0.03 (*n* = 3)	−23.69 ± 0.21 (*n* = 3)	−23.60	0.09
S3	−12.20 ± 0.10 (*n* = 3)	−22.61 ± 0.18 (*n* = 4)	−22.25	0.36
S4	−27.48 ± 0.07 (*n* = 3)	−31.56 ± 0.18 (*n* = 4)	−31.42	0.14

^a^Stable carbon isotopic compositions reported in per mil relative to PDB; ^b^the arithmetic means and standard deviations; ^c^*δ*^13^C values determined by EA/IRMS; ^d^*δ*^13^C values determined by GC/C/IRMS; ^e^*δ*^13^C_MBA_ = −59.42 ± 0.13‰ (*n* = 3, determined by EA/IRMS) *δ*^13^C_TMS_ = −29.96 and was obtained by reacting the same batch of MSTFA with *myo*-inositol standard (*δ*^13^C = −14.65 ± 0.03‰; *n* = 3, determined by EA/IRMS); ^f^on the basis of the mass balance relationship equation (1); ^g^calculated *δ*^13^C_LMT_-measured *δ*^13^C_LMT_; *n*: number of measured times.

**Table 3:** *δ* ^13^C values of microcrystalline cellulose and its combustion particle product levoglucosan under four temperatures.

Combustion temperature (°C)	*δ* ^13^C (‰)			
	Measured cellulose by EA/RMS	Measured LMT by GC/C/IRMS	Calculated levoglucosan^a^	Δ^b^
300	−25.15	−27.27 ± 0.31	−23.24	−1.91
400		−27.34 ± 0.22	−23.42	−1.73
500		−27.55 ± 0.15	−23.93	−1.21
600		−27.40 ± 0.19	−23.56	−1.59

^a^On the basis of equation *δ*^13^C_levoglucosan_ = *f*_LMT_*δ*^13^C_LMT_ − *f*_MBA_*δ*^13^C_MBA_ − *f*_TMS_*δ*^13^C_TMS_, *f*_LMT_ = 5/3, *f*_MBA_ = 1/6, *f*_TMS_ = 1/2, *δ*^13^C_MBA_ = −59.42‰, and *δ*^13^C_TMS_ = −29.96‰; ^b^measured *δ*^13^C_cellulose_ − calculated *δ*^13^C_levoglucosan_.

## Data Availability

The data used to support the findings of this study are available within the article.
